# Utility of Crowdsourced User Experiments for Measuring the Central Tendency of User Performance: A Case of Error-Rate Model Evaluation in a Pointing Task

**DOI:** 10.3389/frai.2022.798892

**Published:** 2022-03-17

**Authors:** Shota Yamanaka

**Affiliations:** Yahoo! JAPAN Research, Yahoo Japan Corporation, Tokyo, Japan

**Keywords:** crowdsourcing, graphical user interface, Fitts'law, user performance models, error-rate prediction

## Abstract

The usage of crowdsourcing to recruit numerous participants has been recognized as beneficial in the human-computer interaction (HCI) field, such as for designing user interfaces and validating user performance models. In this work, we investigate its effectiveness for evaluating an error-rate prediction model in target pointing tasks. In contrast to models for operational times, a clicking error (i.e., missing a target) occurs by chance at a certain probability, e.g., 5%. Therefore, in traditional laboratory-based experiments, a lot of repetitions are needed to measure the central tendency of error rates. We hypothesize that recruiting many workers would enable us to keep the number of repetitions per worker much smaller. We collected data from 384 workers and found that existing models on operational time and error rate showed good fits (both *R*^2^ > 0.95). A simulation where we changed the number of participants *N*_*P*_ and the number of repetitions *N*_repeat_ showed that the time prediction model was robust against small *N*_*P*_ and *N*_repeat_, although the error-rate model fitness was considerably degraded. These findings empirically demonstrate a new utility of crowdsourced user experiments for collecting numerous participants, which should be of great use to HCI researchers for their evaluation studies.

## 1. Introduction

In the field of human-computer interaction (HCI), a major topic is to measure the time needed to complete a given task for (e.g.,) evaluating novel systems and techniques. Examples include measuring a text-entry time (Banovic et al., 2019; Cui et al., 2020), a time to learn a new keyboard layout (Jokinen et al., 2017), and a menu-selection time (Bailly et al., 2016). In these studies, generally, laboratory-based user experiments have been conducted. That is, researchers recruit ten to 20 students from a local university and ask them to use a specified apparatus to perform a task in a silent room. However, researchers are aware of the risk of conducting a user experiment with a small sample size; e.g., the statistical power is weak (Caine, 2016). Therefore, using crowdsourcing services to recruit numerous participants has recently become more common, particularly for user experiments on graphical user interfaces (GUIs), e.g., (Komarov et al., 2013; Matejka et al., 2016; Findlater et al., 2017; Yamanaka et al., 2019; Cockburn et al., 2020).

There are two representative topics for research involving GUIs. The first is designing better GUIs or interaction techniques. In typical user experiments, researchers would like to compare a new GUI or technique with a baseline to demonstrate that a proposed one is statistically better. For this purpose, recruiting numerous participants is effective in finding statistical differences.

The other topic involving GUI experiments is deriving user performance models and empirically validating them. Conventionally, there are two representative metrics for GUI operations to be modeled: time and error rate (Wobbrock et al., 2008). A well-known model in HCI is Fitts' law (Fitts, 1954) to predict the operational time for target pointing tasks, or referred to as *Fitts's law* in some papers (MacKenzie, 2002). In lab-based user experiments to evaluate the model fitness in terms of *R*^2^, university student participants typically join a study and are asked to point to a target repeatedly. For example, researchers set three target distances and three target sizes (i.e., nine task conditions in total), and the participants repeatedly click a target 15 times for each task condition. The average time for these 15 clicks is recorded as the final score for a participant (Soukoreff and MacKenzie, 2004).

In addition to operation times, the importance of predicting how accurately users can perform a task has recently been emphasized (Bi and Zhai, 2016; Huang et al., 2018, 2020; Park and Lee, 2018; Yamanaka et al., 2020; Do et al., 2021). In contrast to measuring the target-pointing times, where the time to click a target can be measured in every trial, the error rate is computed after repeatedly performing a single task condition (15 trials in the above-mentioned case). For example, if a participant misses a target in one trial, the error rate is recorded as 1/15 ×100% = 6.67%; if there are ten participants, one miss corresponds to 0.667% in the end. Because errors can occur by chance, evaluating error-rate models often requires more data (repetitions) for each task condition to measure the central tendency of the error rate. To evaluate the model's prediction accuracy more precisely, researchers have asked participants to perform more repetitions, as it is often difficult to collect numerous participants for lab-based experiments. For example, a previous study on touch-based error-rate models set 40 repetitions for each task condition collected from 12 participants. In this case, one miss corresponded to a 0.208% error rate (Yamanaka and Usuba, 2020).

However, for crowdsourced user experiments with GUIs, researchers cannot set a large number of repetitions per task condition. To enable crowdworkers to concentrate on a given task, it is recommended to set short task completion times, as workers switch to other tasks every 5 min on average (Gould et al., 2016). Hence, forcing a routine GUI operation task that takes, e.g., 40 min (Huang et al., 2018) or 1 h (Park and Lee, 2018; Yamanaka et al., 2020) would be harmful in terms of accurate measurement of the error rates. This could be considered a disadvantage of crowdsourced GUI study. An alternative to increasing the number of repetitions per task condition is simply to recruit more workers. This would enable the error rates to be measured more precisely, which would lead to a good prediction accuracy by the error-rate model (our research hypothesis). Even if the number of repetitions is only ten, utilizing 300 workers would mean that one miss corresponds to 0.033%. This is much more precise than the above-mentioned examples with error rates such as 0.208%.

However, there are several crowdsourcing-specific uncertainties that might affect the user performance results. For example, crowdworkers use different mice, displays, operating systems, cursor speed configurations, and so on; these factors significantly affect the target pointing performance in terms of both time and accuracy (MacKenzie et al., 2001; Casiez and Roussel, 2011). In addition, while studies have shown that the performance model on time (Fitts' law) is valid for crowdsourced data, crowdworkers tend to be more inaccurate than lab-based participants in target pointing tasks (Komarov et al., 2013), where error rates approximately two times higher or more have been observed (Findlater et al., 2017). Therefore, we would avoid claiming that user-performance models validated in crowdsourced studies are always applicable to lab-based controlled experiments. Also, it is not reasonable to interpret that the results such as error rates and operational times are directly comparable with lab-based participants.

Nevertheless, if an error-rate model we test exhibits a good fit (e.g., *R*^2^ > 0.9), HCI researchers would have access to a powerful tool, crowdsourcing, to evaluate their newly proposed error-rate prediction models. Such a result stands to expand the application range of crowdsourcing in HCI; this motivated us to conduct this work. Our contributions are as follows.

- We conducted a crowdsourced mouse-pointing experiment following the Fitts' law paradigm. In total, we recorded 92,160 clicks performed by 384 crowd workers. Our error-rate model showed a good fit with *R*^2^ = 0.9581, and cross-validation confirmed that the model can predict new (unknown) task conditions, too. This is the first study that demonstrates a GUI error-rate model holding to crowdsourced user data.
- We simulated how the number of participants *N*_*P*_ and the number of repetitions per task condition *N*_repeat_ affected the model fitness. We randomly sampled a limited portion of the entire workers (*N*_*P*_ from 10 to 320), and while each worker performed ten trials per task condition, we used only the data for the first *N*_repeat_ trials (from 2 to 10). After testing the model fitness over 1,000 iterations, we found that increasing *N*_*P*_ improved the prediction accuracy as well as increasing *N*_repeat_ could. The effect of *N*_*P*_ and *N*_repeat_ on the fitness was more clearly observed for the error-rate model than the time model, which suggests that crowdsourcing services are more suitable for evaluating novel error-rate models.

This article is an extended version of our previous work presented at the AAAI HCOMP 2021 conference (Yamanaka, 2021b). The points of difference are mainly twofold. First, to analyze the empirical data in more detail, this article newly shows figures that visualize statistically significant differences for the main and interaction effects of independent variables on the outcomes (operational time, click-point variability, and error rate) (see Figures 3, 5, 7). Second, we re-ran the simulation in which the random-sampling was repeatedly performed over 1,000 iterations, while in the conference-paper version we did it over 100 iterations. This larger number of iterations gives us more reliable, less noisy data. We also newly added the standard deviation *SD* values of the model fitness for the 1,000 iterations for the sake of completeness (see Figure 9). Several discussions on these new results, such as comparisons with previous studies regarding model fitness, are also added in this revision.

## 2. Related Work

### 2.1. Time Prediction for Pointing Tasks

**Figure 1:**
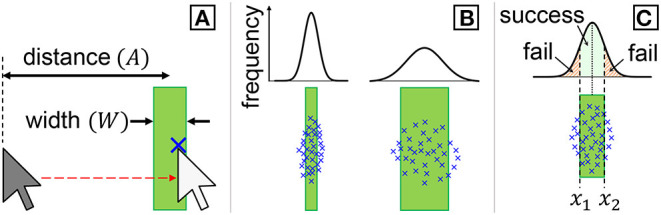
**(A)** We use the Fitts' law paradigm in which users point to a vertically long target. A clicked position is illustrated with an “x” mark. **(B)** It has been assumed that the click positions recorded in many trials distribute normally, and its variability would increase with the target width. **(C)** An error rate is computed based on the probability where a click falls outside the target.

For comparing the sensitivity of time and error-rate prediction models against *N*_*P*_ and *N*_repeat_, we examine a robust time-prediction model, called Fitts' law (Fitts, 1954). According to this model, the time for the first click, or movement time *MT*, to point to a target is linearly related to the index of difficulty *ID* measured in bits:


(1) $$\begin{array}{l} MT=a+b\cdot ID=a+b\cdot \left(\frac{A}{W}+1\right), \end{array}$$

where *a* and *b* are empirical regression constants, *A* is the target distance (or amplitude), and *W* is its width (see Figure 1A). There are numerous formulae for calculating the *ID*, such as using a square root instead of the logarithm or using the effective target width (Plamondon and Alimi, 1997), but previous studies have shown that Equation 1 yields excellent model fitness (Soukoreff and MacKenzie, 2004). Using this Fitts' law, researchers can measure *MT*s for several {*A, W*} conditions, regress the data to compute *a* and *b*, and then predict the *MT* for a new {*A, W*} condition by applying the parameters of {*a, b, A, W*} to Equation 1.

### 2.2. Error-Rate Prediction for Pointing Tasks

Researchers have also tried to derive models to predict the error rate *ER* (Meyer et al., 1988; Wobbrock et al., 2008; Park and Lee, 2018). In practice, the *ER* should increase as participants move faster, and vice versa (Zhai et al., 2004; Batmaz and Stuerzlinger, 2021). In typical target pointing experiments, participants are instructed to “point to the target as quickly and accurately as possible,” which is intended to balance the speed and carefulness to decrease both *MT* and *ER* (MacKenzie, 1992; Soukoreff and MacKenzie, 2004).

In pointing tasks, as the target size decreases, users have to aim for the target more carefully to avoid misses. Accordingly, the spread of click positions should be smaller. If researchers conduct a pointing experiment following a typical Fitts' law methodology, in which two vertically long targets are used and participants perform left-right cursor movements, the click positions would follow a normal distribution (Figure 1B) (Crossman, 1956; MacKenzie, 1992). Formally speaking, a click point is a random variable *X* following normal distribution: *X* ~ *N*(μ, σ^2^), where μ and σ are the mean and standard deviation of the click positions on the *x*-axis, respectively. The click point variability σ is assumed to proportionally relate to the target width, or to need an intercept, i.e., linear relationship (Bi and Zhai, 2016; Yu et al., 2019; Yamanaka and Usuba, 2020):


(2) $$\begin{array}{l} \sigma =c+d\cdot W, \end{array}$$

where *c* and *d* are regression constants. The probability density function for a normal distribution, *f*(*x*), is


(3) $$\begin{array}{l} f\left(x\right)=\frac{1}{\sigma \sqrt{2\pi }}e^{-{\left(x-\mu \right)}^{2}/\left(2\sigma^{2}\right)}. \end{array}$$

If we define the target center as located at *x* = 0 with the target boundary ranging from *x*_1_ to *x*_2_ (Figure 1C), the predicted probability for where the click point *X* falls on the target, *P*(*x*_1_ ≤ *X* ≤ *x*_2_), is


(4) $$\begin{array}{l} P(x_{1}\leq X\leq x_{2})=\int_{x_{2}}^{x_{1}}f(x)dx\\ \;=\frac{1}{2}[\text{erf}(\frac{x_{2}-\mu }{\sigma \sqrt{2}})-\text{erf}(\frac{x_{1}-\mu }{\sigma \sqrt{2}})], \end{array}$$

where erf(·) is the Gauss error function:


(5) $$\text{erf}(z)=\frac{2}{\sqrt{\pi }}\int_{z}^{0}e^{-t^{2}}dt.$$

Previous studies have shown that the mean click point is located close to the target center (μ ≈ 0), and σ is not significantly affected by the target distance *A* (MacKenzie, 1992; Bi and Zhai, 2016; Yamanaka and Usuba, 2020). Given the target width *W*, Equation 4 can be simplified and the *ER* is predicted as


(6) $$\begin{array}{l} ER=1-P(-\frac{W}{2}\leq X\leq \frac{W}{2})=1\\ \;-\frac{1}{2}[\text{erf}(\frac{W/2}{\sigma \sqrt{2}})-\text{erf}(\frac{-W/2}{\sigma \sqrt{2}})]=1-\text{erf}(\frac{W}{2\sqrt{2}\sigma }). \end{array}$$

Similarly to the way Fitts' law is used, researchers measure σ for several {*A, W*} conditions, regress the data to compute *c* and *d* in Equation 2, and then predict the σ for a new {*A, W*} condition. In this way (i.e., using the predicted σ based on a new *W*), we can predict the *ER* with Equation 6 for a new task condition. While there are similar but more complicated versions of this model tuned for pointing tasks in virtual reality systems (Yu et al., 2019) and touchscreens (Bi and Zhai, 2016), to our knowledge, there has been no report on the evaluation of this model for the most fundamental computer environment, i.e., PCs with mice.

### 2.3. Crowdsourced Studies on User Performance and Model Evaluation for GUIs

For target pointing tasks in PC environments, Komarov et al. (2013) found that crowdsourced and lab-based experiments led to the same conclusions on user performance, such as that a novel facilitation technique called *Bubble Cursor* (Grossman and Balakrishnan, 2005) reduced the *MT* compared with the baseline point-and-click method. Yamanaka et al. (2019) tested the effects of target margins on touch-pointing performance using smartphones and reported that the same effects were consistently found in crowdsourced and lab-based experiments, e.g., wider margins significantly decreased the *MT* but increased the *ER*. Findlater et al. (2017) showed that crowdworkers had significantly shorter *MT*s and higher *ER*s than lab-based participants in both mouse- and touch-pointing tasks. Thus, they concluded that crowdworkers were more biased towards speed than accuracy when instructed to “operate as rapidly and accurately as possible.”

Regarding Fitts' law fitness, Findlater et al. reported that crowdworkers had average values of Pearson's *r* = 0.926 with mice and *r* = 0.898 with touchscreens (Findlater et al., 2017). Schwab et al. (2019) conducted crowdsourced scrolling tasks and found that Fitts' law held with *R*^2^ = 0.983 and 0.972 for the desktop and mobile cases, respectively (note that scrolling operations follow Fitts' law well Zhao et al., 2014). Overall, these reports suggest that Fitts' law is valid for crowdsourced data regardless of the input device. It is unclear, however, how the *N*_*P*_ affects model fitness, because these studies used the entire workers' data for model fitting.

The only article that tested the effect of *N*_*P*_ on the fitness of user-performance models is a recent work by Yamanaka (2021a). He tested modified versions of Fitts' law to predict *MT*s in a rectangular-target pointing task. The conclusion was that, although he changed *N*_*P*_ from 5 to 100, the best-fit model did not change. However, because he used all *N*_repeat_ clicks, increasing *N*_*P*_ always increased the total data points to be analyzed, and thus the contributions of *N*_*P*_ and *N*_repeat_ could not be analyzed separately. We further analyze this point in our simulation.

In summary, there is a consensus that a time prediction model for pointing tasks (Fitts' law) shows a good fit for crowdsourced data. However, *ER* data have typically been reported as secondary results when measuring user performance in these studies. At least, no studies on evaluating *ER* prediction models have been reported so far. If we can demonstrate the potential of crowdsourced *ER* model evaluation, at least for one example task (target pointing in a PC environment), it will motivate future researchers to investigate novel *ER* models with less recruitment effort, more diversity of participants, and less time-consuming data collection. This will directly benefit the contribution of crowdsourcing to the HCI field.

## 3. User Experiment

We conducted a traditional cyclic target-pointing experiment on the *Yahoo! Crowdsourcing* platform (https://crowdsourcing.yahoo.co.jp). Our affiliation's IRB-equivalent research ethics team approved this study. The experimental system was developed with the Hot Soup Processor programming language. The crowdworkers were asked to download and run an executable file to perform the experimental task.

### 3.1. Task, Design, and Procedure

**Figure 2:**
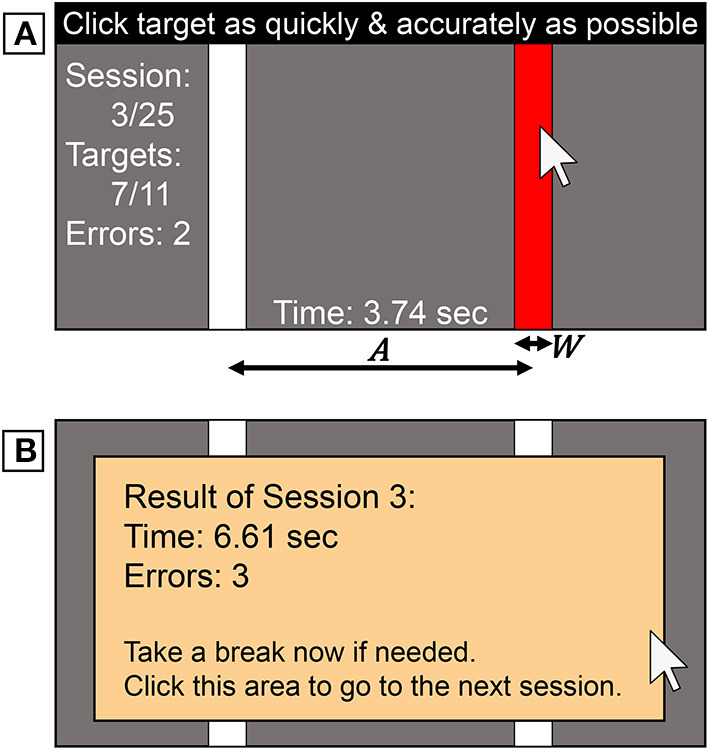
Task stimuli used in the experiment. **(A)** Participants clicked alternately on each target when it was red. **(B)** At the end of a session, the results and a message to take a break were shown.

In the task window (1200×700 pixels), two vertically long targets were displayed (Figure 2A). If the participants clicked the target, the red target and white non-target rectangles switched colors, and they successively performed this action back and forth. If the participants missed the target, it flashed yellow, and they had to keep trying until successfully clicking it. We did not give auditory feedback for success or failure, as not all the participants would have been able to hear sound during the task. A *session* consisted of 11 cyclic clicks with a fixed *A* × *W* condition. The first click acted as a starting signal as we could not measure the *MT*, and thus the remaining ten trials for each session were used for data analysis. After completing a session, the participant saw the results and a message to take a break (Figure 2B).

The experiment was a 3×8 within-subjects repeated-measures design with the following independent variables and levels: three target distances (*A*= 300, 460, and 630 pixels) and eight widths (*W*= 8, 12, 18, 26, 36, 48, 62, and 78 pixels). These values were selected so that the values of *ID* ranged widely from 2.28 to 6.32 bits, which sufficiently covered easy to hard conditions according to a survey (Soukoreff and MacKenzie, 2004). Each participant completed 24 (= 3_*A*_ × 8_*W*_) sessions. The order of the 24 conditions was randomized. Before the first session, to allow the participants to get used to the task, they performed a practice session under a condition with *A* = 400 and *W* = 31 pixels, i.e., parameters that were not used in the actual 24 data-collection sessions. This experimental design was tuned with reference to the author's pilot study; without having a break, the task completion time was 3 min 40 s on average, which meets the recommendation for crowdsourced user experiments (Gould et al., 2016).

The *MT* was measured from when the previous target was successfully clicked to when the next click was performed regardless of the success or failure (MacKenzie, 1992; Soukoreff and MacKenzie, 2004). Trials in which we observed one or more clicks outside the target were flagged as an error. The first left target acted as a starting button, and the remaining ten trials' data were measured to compute *MT*, σ, and *ER*. After finishing all sessions, the participants completed a questionnaire on their age (numeric), gender (free-form to allow non-binary or arbitrary answers), handedness (left or right), Windows version (free-form), input device (free-form), and history of PC use (numeric in years).

### 3.2. Participants and Recruitment

We recruited workers who used Windows Vista or a later version to run our system. We requested no specific PC skills, as we did not wish to limit our collection to only high-performance workers' data. Also, we did not use any *a*-*priori* filtering options, such as the approval-rate threshold, which require additional cost for the crowdsourcing service. We made this decision because, if our hypothesis is supported with a less costly method, it would be more beneficial for future research to recruit many more participants with low cost for obtaining the central tendency of error rates. Still, clear outlier workers who seemed not to follow our instructions (such as performing the task too slowly) were removed when we analyzed the data. As we show later in the simulation analysis, this decision was not problematic because Fitts' law held well even if we analyzed only ten workers' data over 1,000 iterations (i.e., they exhibited typical rapid-and-accurate pointing behavior).

On the recruitment page, we asked the workers to use a mouse if possible. We made this request because, in our simulation analysis, we randomly selected a certain number of participants (e.g., *N*_*P*_ = 10) to examine if the model fitness was good or poor. If these workers used different devices (e.g., six mice, two touchpads, and two trackballs), we might have wondered if a poor model fit was due to the device differences. Nevertheless, to avoid a possible false report in which all workers might answer they used mice, we explicitly explained that any device was acceptable, and then removed the non-mouse users from the analysis.

Once workers accepted the task, they were asked to read the online instructions, which stated that they should perform the task as rapidly and accurately as possible. This was also always written at the top of the experimental window as a reminder (Figure 2A). After they finished all 25 sessions (including a practice session) and completed the questionnaire, the log data was exported to a csv file. They uploaded the file to a server and then received a payment of JPY 100 (~USD 0.92).

In total, 398 workers completed the task, including 384 mouse users according to the questionnaire results. Hereafter, we analyze only the mouse-users' data. The mouse users' demographics were as follows. Age: 16 to 76 years, with *M* = 43.6 and *SD* = 11.0. Gender: 300 male, 79 female, and 5 chose not to answer. Handedness: 24 were left-handed and 360 were right-handed. Windows version: 1 used Vista, 27 used Win7, 8 used Win8, and 348 used Win10. PC usage history: 0 (less than 1 year) to 45 years, with *M* = 21.8 and *SD* = 7.82.

In this study, we do not analyze these demographic data in detail. For example, it has been reported that participants' handedness (Hoffmann, 1997), gender and age (Brogmus, 1991) affect Fitts' law performance. In our simulation, it is possible that the data may be biased; e.g., when we select *N*_*P*_ = 10 workers, they are all males in their 60s. If researchers want to investigate this point, controlling the sampled workers' demographics before executing the simulation is needed.

For mouse users, the main pointing task took 3 min 45 s on average without breaks. With breaks, the mean task completion time was 5 min 42 s, and thus the effective hourly payment was JPY 1,053 (~USD 9.69). Note that this effective payment could change depending on other factors such as the times for reading the instructions and for uploading the csv file.

## 4. Results

### 4.1. Outlier Data Screening

Following previous studies (MacKenzie and Isokoski, 2008; Findlater et al., 2017), we removed trial-level spatial outliers if the distance of the first click position was shorter than half of target distance *A*/2 (i.e., clicking closer to the non-target than the target) to omit clear accidental operations such as double-clicking the previous target. Another criterion used in these studies was to remove trials in which the click position was more than twice of target width 2*W* away from the target center. We did not use this criterion, as we would like to measure error trials even where a click position was ≥(2*W* + 1) pixels away from the target center.

To detect trial-level temporal outliers to remove extremely fast or slow operations, we used the inter-quartile range (*IQR*) method (Devore, 2011), which is more robust than the *mean-and-3*σ approach. The *IQR* is defined as the difference between the third and first quartiles of the *MT* for each session for each participant. Trials in which the *MT* was more than 3×*IQR* higher than the third quartile or more than 3 × *IQR* lower than the first quartile were removed.

For participant-level outliers, we calculated the mean *MT* across all 24 conditions (3_*A*_ × 8_*W*_) for each participant. Then, using each participant's mean *MT*, we again applied the *IQR* method and removed extremely rapid or slow participants. The trial- and participant-level outliers were independently detected and removed.

As a result, among the 92,160 trials (= 3_*A*_×8_*W*_×10_repetitions_×384_workers_), we identified 1,191 trial-level outliers (1.29%). We also found two participant-level outlier workers. While the mean *MT* of all participants was 898 ms and the *IQR* was 155 ms, the outlier workers' mean *MT*s were 1,462 and 1,533 ms. Accordingly, the data from all 480 trials of these two workers were removed (= 3_*A*_×8_*W*_×10_repetitions_×2_workers_). They also exhibited seven trial-level outliers (i.e., there were overlaps). In total, the data from 1,664 trials were removed (1.81%), which was close to the rate in a previous study (Findlater et al., 2017). As a result, we analyzed the remaining data from 90,496 trials.

### 4.2. Analyses of Dependent Variables

After the outliers were removed, the data from 90,496 trials (98.2%) were analyzed. The dependent variables were the *MT*, σ, and *ER*.

#### 4.2.1. Movement Time

We used the Shapiro-Wilk test (α = 0.05) and Q-Q plot to check the normality assumption required for parametric ANOVAs. The *MT* data did not pass the normality test, and thus we log-transformed the data to meet the normality assumption. The log-transformed data passed the normality test, and we used RM-ANOVAs with Bonferroni's *p*-value adjustment method for pairwise comparisons. For the *F* statistic, the degrees of freedom were corrected using the Greenhouse-Geisser method when Mauchly's sphericity assumption was violated (α = 0.05).

**Figure 3:**
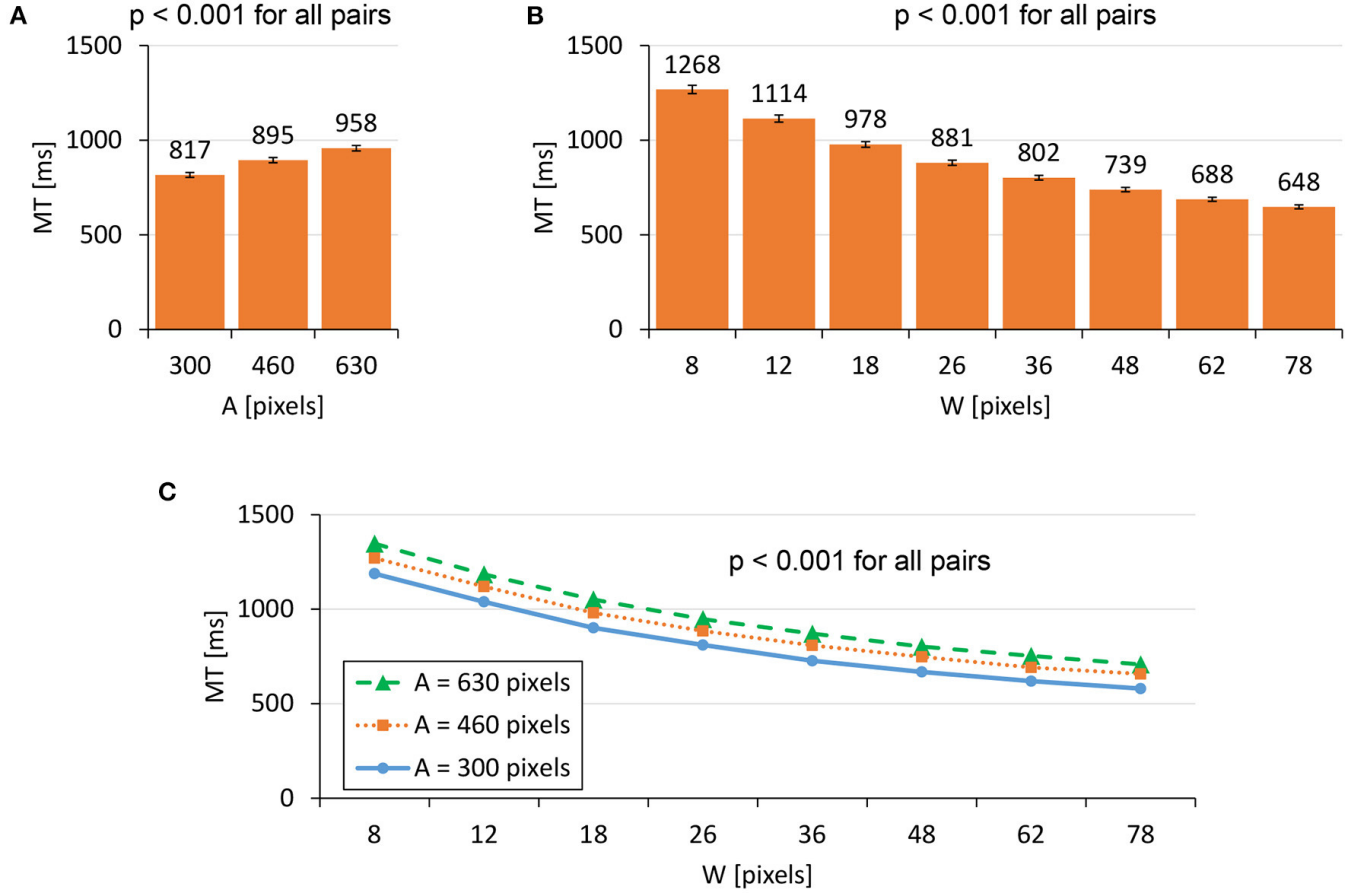
Main effects of **(A)** target distance *A* and **(B)** target width *W* on *MT*. **(C)** The interaction effect of *A*×*W* on *MT*. Error bars indicate 95% confidence intervals.

**Figure 4:**
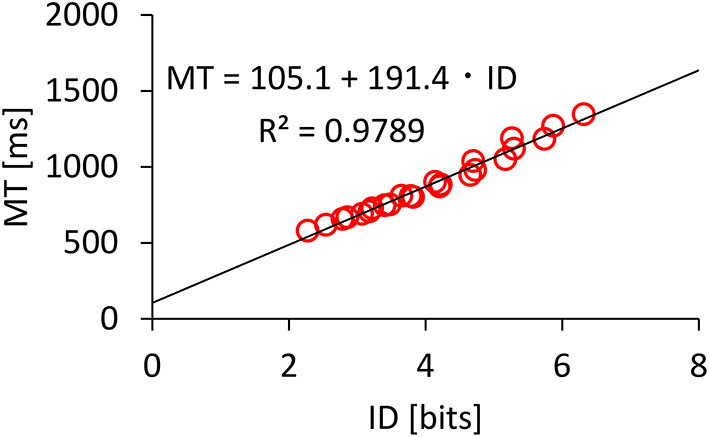
Model fitness results for Fitts' law.

We found significant main effects of *A* (*F*_1.909,727.1_ = 2674, *p* < 0.001, $\eta_{p}^{2}=0.88$) and *W* (*F*_4.185,1595_ = 6813, *p* < 0.001, $\eta_{p}^{2}=0.95$) on *MT*. A significant interaction was found for *A* × *W* (*F*_13.01,4955_ = 14.23, *p* < 0.001, $\eta_{p}^{2}=0.036$). Figure 3 shows that the *MT* increased as *A* increased or *W* decreased. Regarding Fitts' law fitness, Figure 4 shows that the model held well with *R*^2^ = 0.9789. Previous studies using mice have reported that Fitts' law held with *R*^2^ > 0.9 (Plamondon and Alimi, 1997; MacKenzie, 2013), and our dataset was consistent with these results.

#### 4.2.2. Click Point Variability

**Figure 5:**
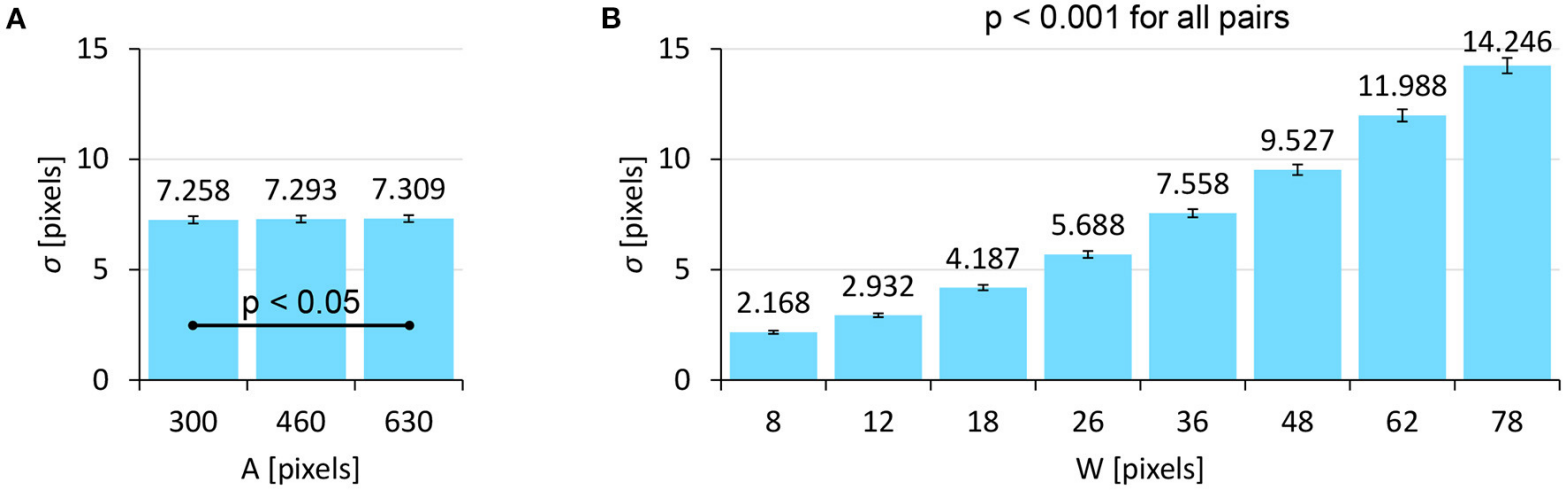
Main effects of **(A)** target distance *A* and **(B)** target width *W* on σ. Error bars indicate 95% confidence intervals.

**Figure 6:**
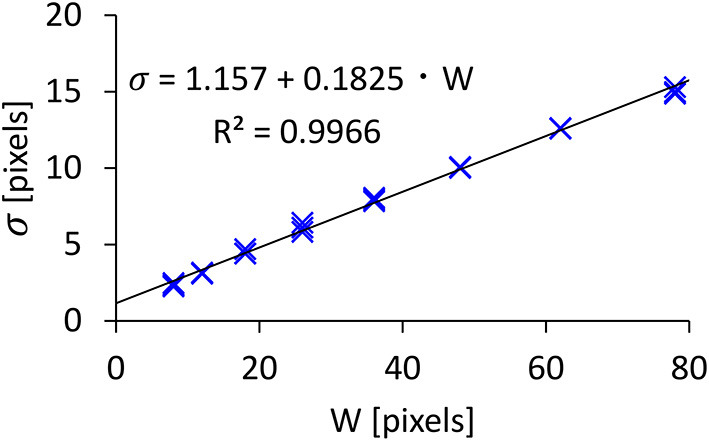
Model fitness results for click point variability.

The σ data and its log-transformed data did not pass the normality test, and thus we used a non-parametric ANOVA with aligned rank transform (Wobbrock et al., 2011) with Tukey's *p*-value adjustment method for pairwise tests. We found significant main effects of *A* (*F*_2,762_ = 3.683, *p* < 0.05, $\eta_{p}^{2}=0.0096$) and *W* (*F*_7,2667_ = 6043, *p* < 0.001, $\eta_{p}^{2}=0.94$) on σ. An interaction of *A*×*W* was not significant (*F*_14,5334_ = 0.8411, *p* = 0.62, $\eta_{p}^{2}=0.0022$). Figure 5 shows that the σ increased as *A* or *W* increased. The model fitness of Equation 2 (σ = *c* + *d* · *W*) was quite high (*R*^2^ = 0.9966), as shown in Figure 6. This fitness was greater than the results in previous studies, e.g., *R*^2^ = 0.9756 (Bi and Zhai, 2013) and *R*^2^ = 0.9763 (Yamanaka and Usuba, 2020) using touchscreens, and *R*^2^ = 0.9931 using a virtual-reality input device (Oculus Touch wireless controller) (Yu et al., 2019).

Our model assumes that σ is not affected by *A*, but the result showed that *A* significantly affected σ. This statistical significance likely comes from the large number of participants. When we checked this in more detail, we found that the effect size of *A* was quite small compared with *W* ($\eta_{p}^{2}=0.0096$ vs. 0.94, respectively), and the mean σ values for *A*= 300, 460, and 630 pixels were 7.258, 7.293, and 7.309 pixels, which fall within a 0.051-pixel range (<1%). In contrast, the σ values varied from 2.168 to 14.25 pixels due to *W* (i.e., a 557% difference). While we plotted 24 points (3_*A*_ × 8_*W*_) in Figure 6, it looks as though there were only eight points, as the three σ values for the three *A*s were almost the same and thus they overlapped.

#### 4.2.3. Error Rate

**Figure 7:**
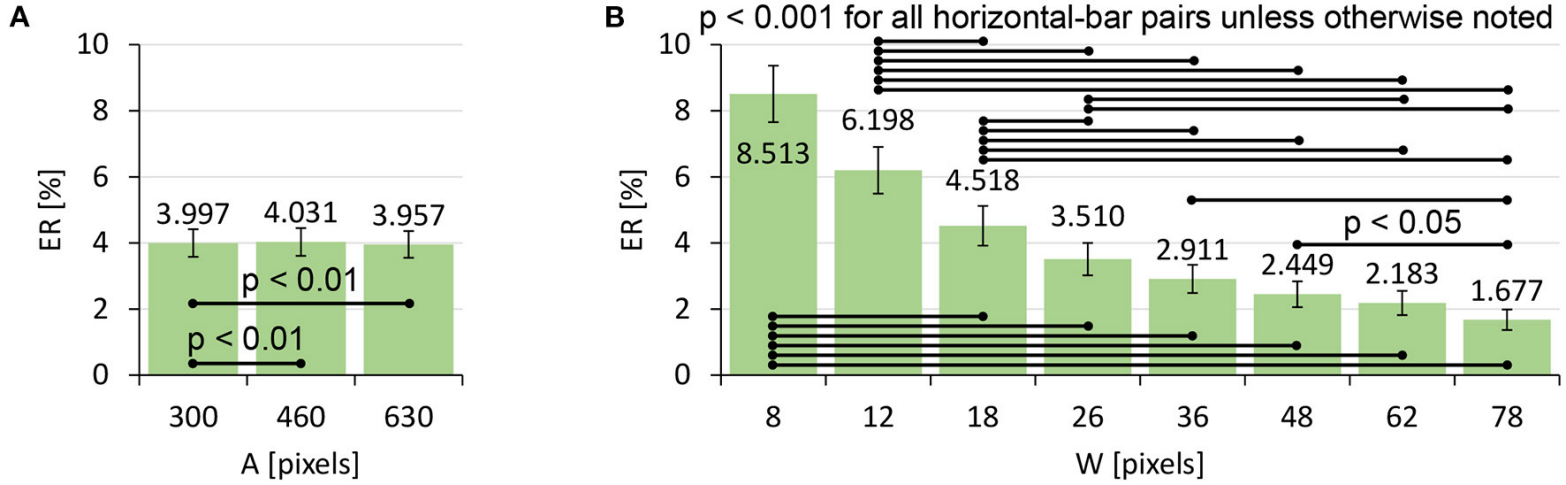
Main effects of **(A)** target distance *A* and **(B)** target width *W* on *ER*. Error bars indicate 95% confidence intervals.

The *ER* data and its log-transformed data did not pass the normality test, and thus we again used a non-parametric ANOVA with aligned rank transform. We found significant main effects of *A* (*F*_2,762_ = 6.732, *p* < 0.01, $\eta_{p}^{2}=0.017$) and *W* (*F*_7,2667_ = 96.90, *p* < 0.001, $\eta_{p}^{2}=0.20$) on *ER*. An interaction of *A* × *W* was not significant (*F*_14,5334_ = 1.627, *p* = 0.064, $\eta_{p}^{2}=0.0043$). Figure 7 shows that the *ER* decreased as *W* increased, while *A* did not exhibit a clear tendency to increase/decrease the *ER*.

**Figure 8:**
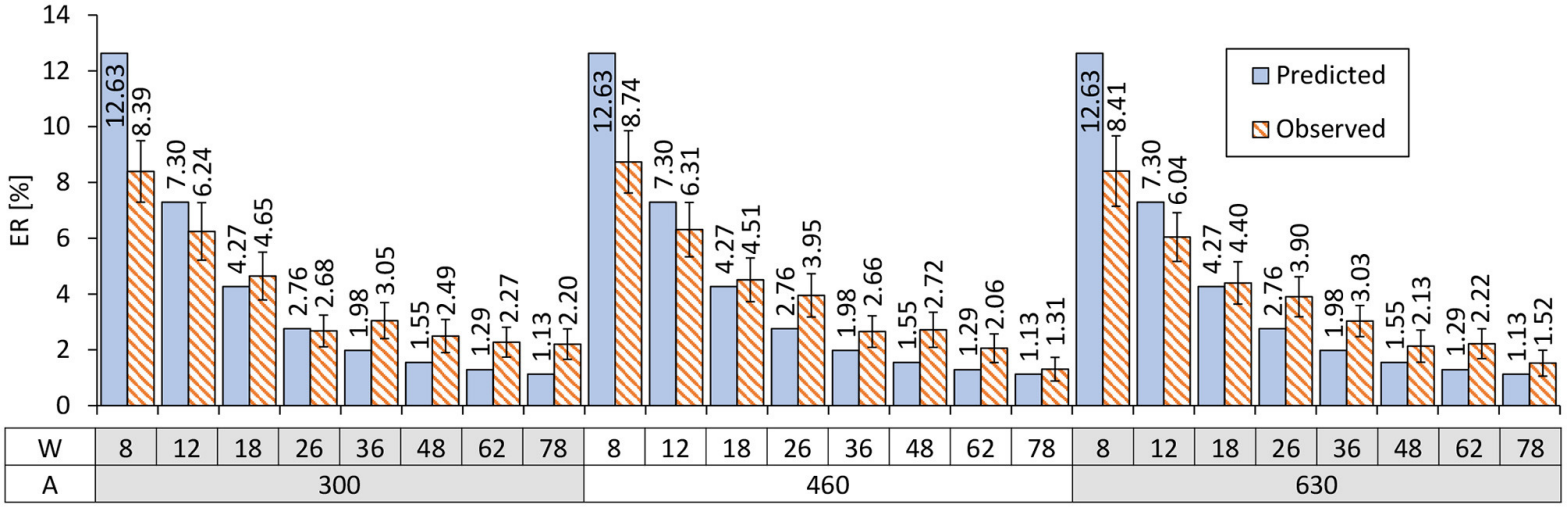
Comparison of the predicted vs. observed *ER*s. Error bars indicate 95% confidence intervals.

Using Equations 2 and 6, we can predict the *ER*s based on given *W* values. The predicted and actually observed *ER*s are shown in Figure 8. The worst prediction error was 4.235 points in the case of (*A, W*) = (300, 8). As a comparison, previous studies on touch-based pointing tasks have reported that the prediction error for *W*= 2.4-mm targets was 9.74 points (Bi and Zhai, 2016) and that for 2-mm was 10.07 points (Yamanaka and Usuba, 2020). While a direct comparison with touch operations is not particularly fruitful, the tendency that prediction errors increase for smaller *W*s is consistent between the previous studies and ours.

To formally evaluate our model's prediction accuracy, we computed the following three fitness criteria. The correlation between predicted vs. observed *ER*s was *R*^2^ = 0.9581. The mean absolute error *MAE* was 1.193%. The root mean square error *RMSE* was 1.665%. In addition, to evaluate the prediction accuracy for new (unknown) task conditions, we ran a leave-one-(*A, W*)-out cross-validation. The three criteria for the *ER* prediction were *R*^2^ = 0.9529, *MAE* = 1.272%, and *RMSE* = 1.814. The worst prediction error was 4.805 points. These results indicate that, even for researchers who would like to predict the *ER* for a new task condition based on previously measured data, the prediction accuracy would not be considerably degraded.

## 5. Simulation

Although our *N*_repeat_ (10) was not large compared with previous studies on error-rate prediction models due to the time constraint for crowdsourcing, we hypothesized that increasing *N*_*P*_ would improve the model fitness. We also wonder how the model fitness changes when *N*_repeat_ is much smaller, which further shortens the task completion time for workers. For example, if it were 5, the average task completion time would be 2 min 51 s including breaks (i.e., half of 5 min 42 s). Note that *N*_repeat_ must be greater than 1 to compute the standard deviation σ.

We randomly selected *N*_*P*_ workers' data from the 384 mouse users by changing *N*_*P*_ from 10 (typical lab-based experiments) to 320 by doubling it repeatedly. The *N*_repeat_ changed from 2 to 10; if it was 2, we used only the first two repetitions' data and the subsequent eight trials were removed. Outlier detection was run in the same manner as if we had conducted an experiment newly with *N*_*P*_ workers. Then, we analyzed the *R*^2^ values for Equations 1 (Fitts' law), 2 (click point variability σ), and 6 (*ER*). To handle the randomness to select *N*_*P*_ workers, we ran this process over 1,000 iterations and computed the mean and *SD* values of the *R*^2^s for each of *N*_*P*_ × *N*_repeat_.

**Figure 9:**
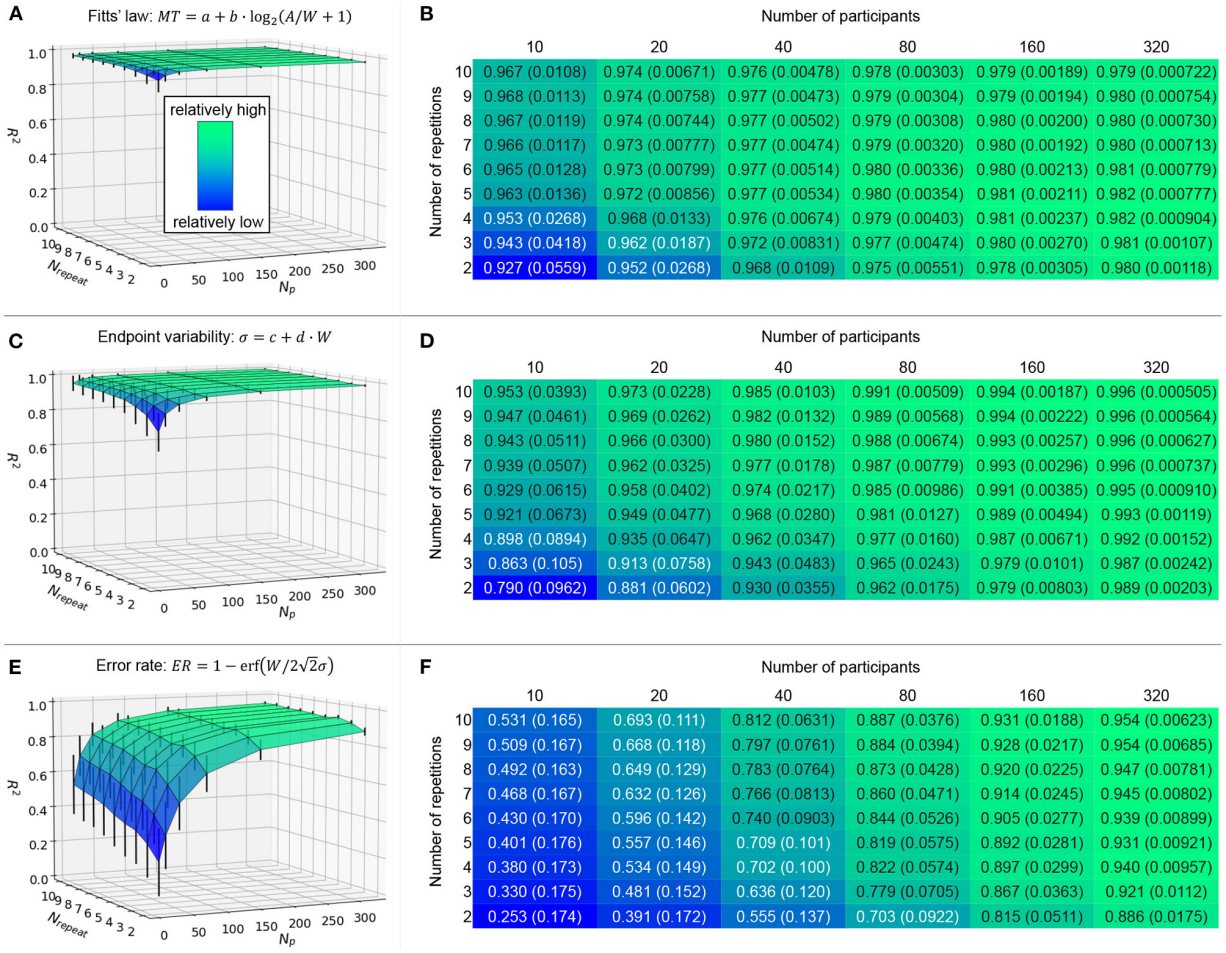
Simulation results on mean (and *SD*) model fitness in *R*^2^ by changing *N*_*P*_ and *N*_repeat_ over 1,000 iterations. Error bars indicate 1*SD*.

The results are shown in Figure 9. First, we can visually confirm that the time prediction model (A) showed the flattest fitness compared with the other two models (C and E). The *R*^2^ values were consistently over 0.92, and after we collected 20 participants or measured four repetitions, *R*^2^ was over 0.95 (B). This result supports the decision of previous studies' lab-based experiments that recruited ten to 20 participants to examine Fitts' law. While repeating 15 to 25 trials per task condition has been recommended (Soukoreff and MacKenzie, 2004), our results show that a much smaller number of repetitions will suffice.

For the click point variability, as (C) shows, the model fitness was relatively worse only when both *N*_*P*_ and *N*_repeat_ are small. The increase in either *N*_*P*_ or *N*_repeat_ can resolve this. For example, by collecting *N*_*P*_ ≥ 80 workers or repeating ten trials, we obtain *R*^2^ > 0.95.

Lastly, for the error-rate model, the fitness was affected by *N*_*P*_ and *N*_repeat_ most drastically, as shown in (E). Particularly for small *N*_*P*_ values such as 10 and 20, the *R*^2^ values were less than 0.70 (F), which is a unique result compared with the other two models that always showed much greater *R*^2^ values in (B) and (D). If we fully use ten repetitions and would like to obtain a certain value of the model fitness (such as *R*^2^ > 0.9), collecting 160 participants is sufficient—more precisely, when we tested *N*_*P*_ from 80 to 160 (step: 1), *N*_*P*_ = 96 achieved mean *R*^2^ = 0.9017 > 0.9 for the first time (*SD* = 0.03208).

Figures 9E,F demonstrates that increasing *N*_*P*_ can be a viable alternative to increasing *N*_repeat_ to obtain a higher prediction accuracy for this error-rate model. Suppose we have a case where researchers want to set a smaller *N*_repeat_ such as 3 instead of 10 due to (e.g.,) asking workers to answer more questionnaire items after the task. Even for this case, by collecting *N*_*P*_ = 320 workers, the model would fit to the data with *R*^2^ > 0.9 in our data. Hence, although the task completion time for crowdsourced user experiments should not be too long (Gould et al., 2016), the easy recruitment for crowdsourcing enables researchers to measure the central tendency of error rates. This benefit of crowdsourcing is more critical for error-rate models than time-prediction models, as we demonstrated here, which has never been empirically reported before.

When *N*_*P*_ or *N*_repeat_ was large, the error bars for model fitness (the *SD* values of *R*^2^ over 1,000 iterations) were small for all models we examined (see Figure 9). This is because the same workers' data were more likely to be selected as the number of measured data points increased, and thus the variability in model fitness became small. In other words, when the number of data points was small, the model fitness depended more strongly on the choice of worker group and their limited trials. This effect of small *N*_*P*_ or *N*_repeat_ values on the large fitness variability was more clearly observed for the *ER* model (Figures 9E,F). Therefore, it is possible that the *ER* model will exhibit a quite low *R*^2^ value when *N*_*P*_ or *N*_repeat_ was small, and at the same time, a much higher *R*^2^ value might also be found by chance. This result shows that the *ER* is relatively not robust against the small number of data points.

In comparison, even when *N*_*P*_ or *N*_repeat_ was small, the error bars of the *MT* and σ models were smaller (Figures 9A,C). In particular, because the mean *R*^2^ values of the *MT* model were already high (>0.92), there remains a limited space to exhibit much lower or higher *R*^2^s, and thus the *SD* values could not be large. This demonstrated the robustness of the operational time prediction using Fitts' law.

## 6. Discussion

### 6.1. Benefits and Implications of Using Crowdsourcing for Error-Rate Model Evaluation

In this study, we explored the potential of crowdsourcing for evaluating error-rate prediction models on GUIs. As one of the most fundamental operations, we utilized a Fitts' law task for its well-structured methodology. The results obtained from 384 crowdworkers showed that the models on Fitts' law and the click point variability fit well to the empirical data with *R*^2^ = 0.9789 and 0.9966, respectively, as shown in Figures 4, 6. Using the predicted σ values based on *W*, we then predicted the *ER*s for each *A* × *W* condition, which yielded the correlation between predicted vs. observed *ER*s of *R*^2^ = 0.9572. The other metrics (*MAE* and *RMSE*) and the cross-validation also showed the good prediction accuracy of the model. On the basis of these results, in addition to the time-prediction model, we empirically demonstrated the first evidence that an error-rate model held well even for crowdsourced user experiments, even though it has been cautioned that crowdworkers are more error-prone in GUI tasks (Komarov et al., 2013; Findlater et al., 2017).

The simulation to alter *N*_*P*_ and *N*_repeat_ showed that the prediction accuracy of the error-rate model became better when either of these values was larger. This effect was more clearly observed for the error-rate model than the time- and click-point-variability models. In particular for the time model, the prediction accuracy reached close to the upper limit (*R*^2^ = 1) even when the *N*_*P*_ and *N*_repeat_ were not large, such as the *R*^2^ > 0.95 exhibited by ten workers performing four repetitions (Figure 9B). This suggests that the advantage of crowdsourcing in terms of its easy recruitment of numerous workers is not so critical. In comparison, for the error-rate model, increasing the *N*_*P*_ was still effective for *N*_*P*_ ≥ 160.

Because the error rate is computed on the basis of occasionally occurring operations (clicking outside the target), researchers need more data to measure the theoretical value. Thus, our result, i.e., that collecting more data would lead to the theoretical value that a model estimates, is intuitive, but it has never been empirically demonstrated until now. Finally, our research hypothesis, “instead of increasing the number of repetitions per task condition, recruiting more workers is another approach to measure the error rates precisely, which will lead to a good prediction accuracy by the error-rate model,” was supported. This is a motivating finding for future studies on evaluating novel error-rate models through crowdsourced user experiments.

Note that, we compared the sensitivity of time and error-rate models against *N*_*P*_ and *N*_repeat_, but our purpose here was not to claim that (e.g.,) Fitts' law is a better model than the error-rate model. As described in the introduction, an *MT* is measured in every trial and then averaged after completing a session consisting of *N*_repeat_ trials, but an *ER* is computed after each session. Due to this difference, surmising that *the error-rate model is inferior* is not appropriate. Although more participants are needed to obtain a good fitness comparable with Fitts' law, which could be a limitation of the error-rate model, it does not necessarily mean that the model is wrong or inaccurate. Collecting numerous participants can avoid reaching such a mistaken conclusion. This point about making a conclusion based on an experiment with small sample size has been made before (Kaptein and Robertson, 2012; Caine, 2016), and our results again support the importance of a large sample size. Using crowdsourcing for error-rate model evaluation is a straightforward way to enable the recruitment of hundreds of participants with a reasonable time period, cost, and effort by researchers, which enhances the contribution of crowdsourcing to an undeveloped use application.

### 6.2. Limitations and Future Work

Our claims are limited to the task we chose and its design. We emphasized the usefulness of crowdsourced user experiments for error-rate model evaluation, but we only tested a GUI-task model implemented with mice following the Fitts' law paradigm. Within this scope, we limited the task design to horizontal movements where the effect of target height was negligible. We assume that modified models can predict *ER*s for more realistic targets such as pointing to circular targets (Bi and Zhai, 2016; Yamanaka and Usuba, 2020), but this needs further investigation in the future.

The model we examined was for selecting static targets, while recently models for more complicated tasks have been proposed, including those for pointing to automatically moving targets (Lee et al., 2018; Park and Lee, 2018; Huang et al., 2019), temporally constrained pointing such as rhythm games (Lee and Oulasvirta, 2016; Lee et al., 2018), and tracking a moving target (Yamanaka et al., 2020). We assume that the benefit of using crowdsourcing services to recruit numerous participants can be observed in these complicated tasks more clearly than our 1D pointing task. For example, pointing to a circular moving target needs more task parameters, such as the initial target distance *A*, its size *W*, movement speed *V*, and movement angle θ (Hajri et al., 2011; Huang et al., 2019). Because there are more task-condition combinations than 1D-target pointing, it is difficult to ask the participants to perform many repetitions per task condition, while recruiting numerous workers is easy in crowdsourced user studies. Investigating error rates in text input tasks is another important topic in the HCI field (Banovic et al., 2019; Cui et al., 2020) and would be a potential objective for crowdsourced user experiments.

A technical limitation specifically for our GUI-based experiment was that we could not check if workers really followed the given instruction, such as using mice and operating as rapidly and accurately as possible. For example, we fully trust the questionnaire results on the workers' devices. However, some mouse-users might use touchpads in actuality, as we had instructed to use mice. Similar concerns have been reported before: for touch pointing tasks with smartphones, researchers could not confirm whether workers tapped a target with their thumb as instructed (Yamanaka et al., 2019). Some other crowdsourcing platforms support an option that task requesters can ask workers to shoot a video when they perform a task, e.g., *UIScope* (http://uiscope.com/en). Still, this would create heavier workloads for both the workers and the experimenters. While these issues could not be completely removed at this time, if they were resolved in the future, the contribution to HCI would be significant.

## 7. Conclusion

We ran a crowdsourced user experiment to examine the benefits of recruiting numerous participants for evaluating an error-rate prediction model in a target pointing task, which is one of the most fundamental operations in PC usage. By analyzing the data obtained from 384 workers, we found that our model held well with *R*^2^ > 0.95. Cross-validation also supported the good prediction accuracy to the unknown task conditions. In addition, when we randomly selected a limited portion of the entire workers from *N*_*P*_ = 10 to 320 and used only a limited number of trial repetitions from *N*_repeat_ = 2 to 10, we found that the time prediction model (Fitts' law) reached *R*^2^ > 0.95 even if both of these values were small, while the error-rate model showed quite low fitness in that case. Thus, we empirically demonstrated that using crowdsourcing services for recruiting many participants is more clearly beneficial for evaluating the error-rate prediction model. Our findings should enhance the contribution of crowdsourcing in the HCI field.

## Data Availability Statement

The datasets presented in this article are not readily available because the dataset used in this article is allowed to be open only in its statistically analyzed state (e.g., mean and standard deviation), and thus the raw dataset is not publicly available. Requests to access the datasets should be directed to SY, syamanak@yahoo-corp.jp.

## Ethics Statement

The studies involving human participants were reviewed and approved by Yahoo JAPAN Research's IRB-equivalent research ethics team. The participants provided their written informed consent to participate in this study.

## Author Contributions

SY has done all tasks required for preparing this article, including software development, data analyses, figure creation, and writing manuscript.

## Conflict of Interest

SY is employed by Yahoo Japan Corporation.

## Publisher's Note

All claims expressed in this article are solely those of the authors and do not necessarily represent those of their affiliated organizations, or those of the publisher, the editors and the reviewers. Any product that may be evaluated in this article, or claim that may be made by its manufacturer, is not guaranteed or endorsed by the publisher.
